# Development of Simplified and Efficient Sample Preparation Methods for the Analysis of Problem Material within the Diesel Fuel Delivery System

**DOI:** 10.1021/acsomega.3c03577

**Published:** 2023-09-25

**Authors:** Molly Wilson, Julie M. Herniman, Jim Barker, G. John Langley

**Affiliations:** † School of Chemistry, University of Southampton, Southampton SO17 1BJ, U.K.; ‡ Innospec Inc., Ellesmere Port, Cheshire CH65 4EY, U.K.

## Abstract

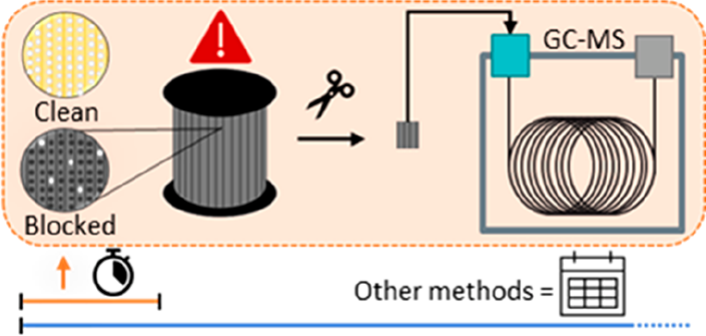

A new approach for the analysis of diesel engine fuel filters has been developed. This method involves minimal to no sample preparation, allowing rapid and unbiased analysis of diesel fuel filters. In recent years, diesel fuel filter plugging incidences have increased in parallel with changing emissions legislation. Fuel filter blockages can result in increased emissions, reduced efficiency, and engine failure. It is not fully understood why fuel filter blockages occur; as a result, there has been an international increase in research into the cause of fuel filter plugging. The method discussed in this paper utilizes a thermal desorption (TD) style sample introduction technique that can be used in conjunction with gas chromatography–mass spectrometry (GC-MS) and presents a fast, simple, and more sustainable approach to the analysis of fuel filters. When required, an efficient and straightforward sample cleanup process was developed and was used to simplify and improve confidence in the data identification and assignment; this method is up to three orders of magnitude faster than some procedures adopted in the literature. Further complementary analytical techniques, such as ultrahigh-performance supercritical fluid chromatography–mass spectrometry (UHPSFC-MS) and high-resolution GC-MS, were used to access additional sample-specific information. This new approach has been successful in the identification of problematic materials deposited on blocked fuel filters, concurrent with recent research. This information can aid in the development of mitigation strategies to combat fuel filter plugging.

## Introduction

Recent legislation regarding vehicle emissions has been shaped by environmental concerns coupled with a consumer-driven focus relating to the efficiency of diesel vehicles.^1^ This has led to a mandate for a reduction in emissions and an increase in engine efficiency. Manufacturers have had to make significant changes to the composition of fuel and engine components to meet these regulations.^1,2^ This includes the introduction of biodiesel as a renewable fuel source as well as advances in the design of fuel-injection equipment (FIE).^3^ The EURO standard regulations were implemented in Europe in 1992^4^ and have been through several iterations with increasingly stringent specifications.^5^ The most recent EURO 6 standards have seen increased use of high-pressure common rail injection systems (HPCR) and improved aftertreatment systems.^6^ However, in parallel with the changing legislation, there has been an increased incidence of blockages within the fuel delivery system of some diesel engines.

Blockages are caused by a buildup of insoluble material within the fuel delivery system and are often associated with the fuel filter. A fuel filter is designed to remove particulate matter from the fuel before it is delivered to the engine but, in recent years, fuel filters are failing before their expected lifetime.^7−9^ Blockages in the filter restrict fuel flow to the engine and subsequently lead to poor engine performance (e.g., reduced engine efficiency and increased vehicle emissions, etc.) or complete engine failure due to fuel starvation. Primary fuel filters are made of fine porosity material such as paper or cloth and filter particles larger than 5 μm. Widespread use of HPCR FIE has resulted in a lower tolerance for insoluble material, and this is owing to the small clearances required for optimum operation of the FIE. Therefore, fuel filters with reduced apertures (2 μm) have been employed to protect the sensitive FIE.^9^

Diesel fuel is a naturally complex material, and the base fuel is a crude oil distillate mostly consisting of hydrocarbons between C10 and C20 including paraffins, isoparaffins, olefins, naphthenes, and aromatics (PIONA).^10^ Additive packages are used to increase the performance and maintain long-term engine efficiency; they include cold flow improvers, corrosion inhibitors, and lubricity improvers, further adding to the complexity of the fuel. Current legislation also now requires biodiesel to be blended with petrodiesel (EN590 in the U.K. and Europe, ASTM D7467 in the United States of America (USA)) to introduce a sustainability aspect of the fuel.^11,12^

Biodiesel is a renewable fuel stock consisting of fatty acid esters, typically methyl esters, manufactured via a transesterification reaction of vegetable oils.^13^ Biodiesel contains fatty acid methyl esters (FAME) with hydrocarbon chain lengths similar to those of petrodiesel and is therefore suitable for blending with diesel. Standard specifications such as ASTM D6751 and EN 14214 have been implemented in the USA and Europe, respectively, to ensure the quality of the biodiesel produced.^14^ Despite this testing, the inclusion of biodiesel within the fuel has been linked to fuel filter plugging.^8,9^

Traditionally, biodiesel feedstock sources can include plant material such as rapeseed, soybean, and palm or alternatively animal fats.^15^ More recently, used cooking oil (UCO) has also been used as a feedstock for biodiesel, this has additional benefits of reducing waste by recycling the UCO and eliminating the issue with potential competition with food production associated with other feedstocks.^16^

Across the diesel industry, filterability issues are not yet fully understood due to investigative challenges and limited research on the subject.^7,17^ Although it is well-known, biodiesel is a suspected contributing factor.^9,17,18^ Trace components in the biodiesel are thought to contribute to the formation of insoluble materials that lead to the blockages,^7^ and these can include naturally occurring chemicals found in biodiesel feedstock such as sterol glucosides (SGs) and/or products formed via incomplete or poor biodiesel manufacture such as saturated monoacylglycerides (SMGs) and glycerol.^9,12,17^ However, the source and identity of some trace species are still unknown. It is possible that these trace components cause issues independently or as part of a cumulative process.^7^

Biodiesel as a potential source of fuel filter plugging is a complex issue owing to the multifaceted and poorly understood nature of insoluble material production.^17^ Biodiesel is prone to oxidation due to the increased oxygen content, and this is exacerbated in feedstocks with high levels of unsaturated sites.^18^ Oxidative degradation of biodiesel produces unwanted products that may have deleterious effects. Water present in the fuel as a result of improper handling or condensation can enable microbial growth. Microorganisms can then feed on components in the fuel expediting degradation processes.^18,19^ It is well-documented that fluctuations in temperature have an effect on the solubility of FAME species as well as trace components such as SMGs.^7,9,17,20^ Long-term storage of biodiesel can result in an accumulation of problematic species, potentially amplifying the issues listed above. It is essential to identify these problematic components in fuel and filter material to give an indication of the source of blockages; this information can subsequently be used to develop mitigation strategies to prevent these blockages from occurring. Although diesel fuel is subjected to rigorous testing procedures that ensure quality, several studies have highlighted filterability issues with fuels that meet international standards.^7,17,20^ Specifically, Heiden et al. and Cardeo et al. saw the formation of insoluble material within the fuel at temperatures above the measured cloud point.

Recent publications have identified a number of common problematic species in blocked diesel engine fuel—these include SGs, SMGs, glycerol, metal carboxylates, and fatty acid sterol esters (FASEs) among others.^7,17,19,21,22^ The scale of the problem is evidenced by a rise in the number of publications regarding blocked fuel filters.^7−9,17,19−24^ In some cases, the techniques involve multiple stages that result in complex and time-intensive sample preparation steps.^17,23^

Csontos et al. have published several papers related to the analysis of fuel filters where they used different analytical techniques, including Fourier transform infrared spectroscopy (FTIR), X-ray fluorescence (XRF), and gas chromatography–mass spectrometry (GC-MS).^21,23,24^ The blocked filters were found to contain glycerol, sterols, and metal soaps. Heiden et al. have also analyzed fuels and blocked filters; their findings mimicked those of Csontos et al. with contaminants such as SMGs, SGs, and glycerol identified.^17^ They also noted that all but one of the fuels contained these contaminants below the defined acceptable limits specified by international testing bodies such as ASTM. The fuel filters were analyzed using GC-FID, with GC-MS used to determine the concentrations of the contaminants within the filters. The related sample preparation was complex and time-intensive involving solid-phase extraction, multiple solvent extractions at −19 °C, and the use of nitrogen to remove excess solvent, with this entire process taking approximately 2 weeks per sample.

Csontos et al. sample preparation methods were less time-intensive but still taking between 2 and 3 h. The process involved washing the fuel filters with cyclohexane to remove excess fuel matrix and then leaving the sample to soak in the solvent for 30 min and was repeated five times. Finally, the sample was washed with methanol to extract polar species and centrifuged before GC-MS analysis.^23^

The GC-MS fuel filter analysis approach discussed herein requires minimal sample preparation and utilizes a thermal desorption (TD) sample introduction technique. The method takes approximately 30 min from sample arrival to results (see Experimental Section for details). This is up to 10 times faster than the method used by Csontos et al. and 1000 times faster than the approach taken by Heiden et al.

## Experimental Section

### Chemicals and Samples

**Figure 1:**
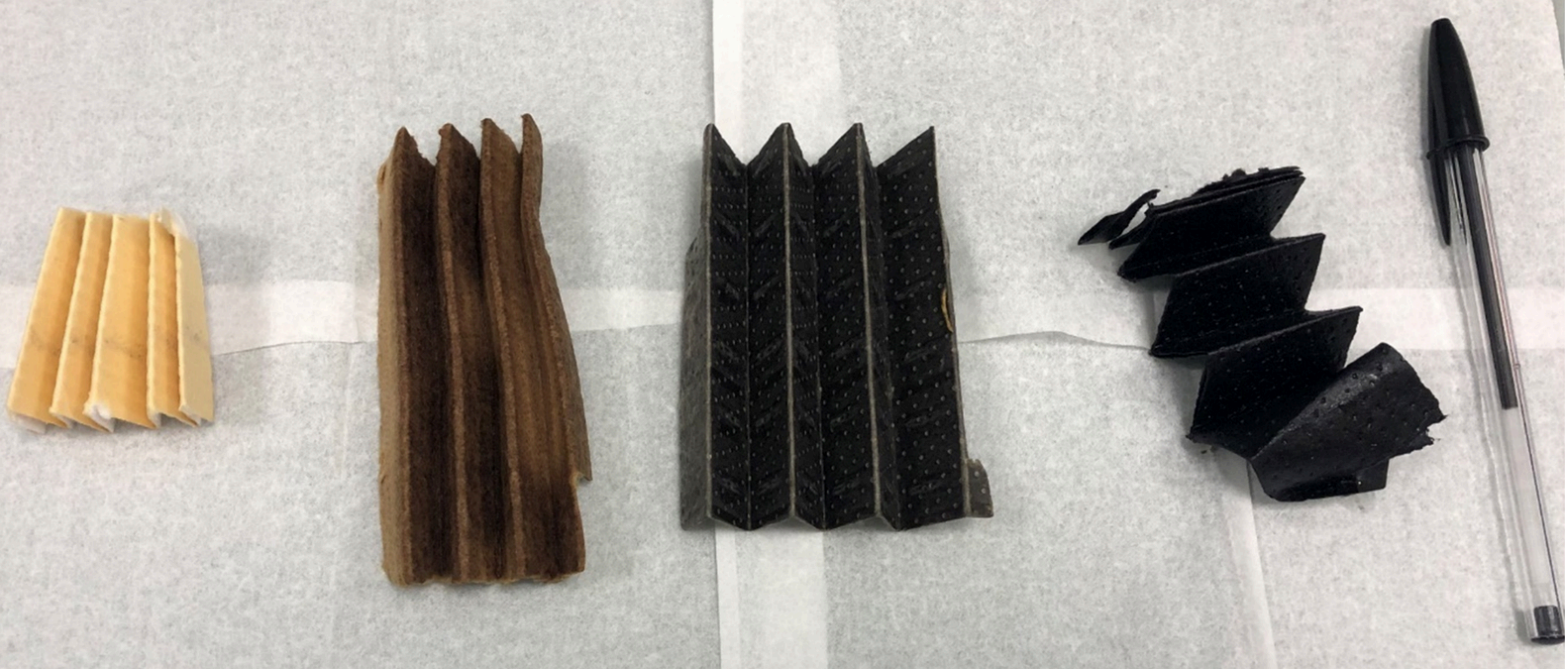
Examples of some of the different fuel filters analyzed, from lightly soiled on the left to heavily soiled on the right. Pen for size reference.

Methanol (liquid chromatography–mass spectrometry (LC-MS) grade) and dichloromethane (high-performance liquid chromatography (HPLC) grade) were purchased from Thermo Fisher Scientific (Loughborough, U.K.). The fuel filters were obtained from scenarios in which the use of petrodiesel/biodiesel fuel blends had subsequently caused fuel filter plugging (Innospec Ltd., Ellesmere Port, U.K.). Some examples of fuel filters are shown in Figure 1.

### TD-Style Sample Introduction Preparation

**Figure 2:**
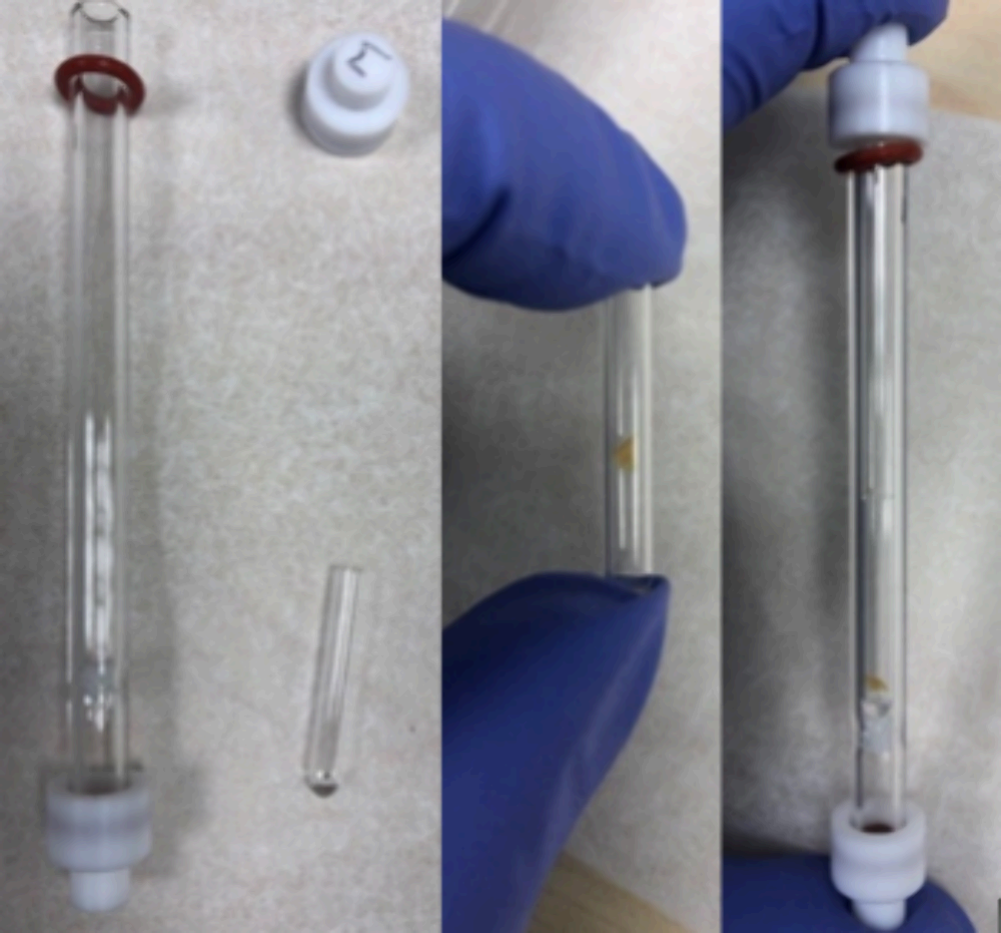
GC liner with caps and microvial (left), microvial containing small section (1 mm^2^) of fuel filter paper (middle), and microvial containing fuel filter inside the GC liner with caps (right).

The instrumentation used in this experiment was a LECO Pegasus BT 4D GCxGC-TOFMS, equipped with an OPTIC multimode inlet system that allows different sample introduction techniques such as liquid, headspace, thermal desorption, etc. TD-style sample introduction was used to analyze fuel filter material, where a small section of fuel filter (approximately 1 mm^2^) was placed into a microvial and then placed into a GC liner (see Figure 2). The liner assembly was inserted into the GC injector unit and rapidly heated from 50 to 210 °C at 6 °C/s and held at 210 °C until the end of the GC-MS acquisition.

### Solvent Extraction

Two small sections (approximately 1 cm^2^) of the fuel filter were cut out and subsequently washed with different solvents, one with dichloromethane (DCM) (3 mL) and one with methanol (3 mL). After 15 min, each filter paper was retrieved and allowed to dry. The solvent extracts were diluted by a factor of 10 prior to GC-MS analysis. A smaller piece (approximately 1 mm^2^) of each of these filter-paper sections was then cut out from the 1 cm^2^ section for analysis by TD-style sample introduction.

### GC-MS

1D GC-MS analysis was performed using a LECO Pegasus BT 4D GCxGC-TOFMS equipped with a Rxi-5SilMS capillary column (Restek), 30 m x 0.25 mm inner diameter, 0.25 μm film thickness; and a Rxi-17SilMS capillary column (Restek) 1.00 m × 0.25 mm inner diameter, 0.25 μm film thickness. Helium was used as carrier gas at a flow rate of 1 mL/min, and a ramped temperature program was used starting at 35 °C, held for 2 min, and then increased at a ramp rate of 20 °C/min to 300 °C and held for 4 min. Analyses using the TD-style approach used a 400:1 split. Samples introduced via liquid injection (1 μL injection volume) used a 50:1 split; the OPTIC injector was heated from 45 to 260 °C at 6 °C/s.

70 eV electron ionization (EI) mass spectra were collected over a range of *m*/*z* 40 to 550 at an acquisition rate of 10 spectra/s with a 200 s solvent delay. Analytes were identified by comparing the EI mass spectrum to the National Institute of Standards and Technology (NIST) mass spectral library. LECO ChromaTOF version 1. 2. 0. 6 software was used to control the chromatography and mass spectrometry methods as well as to acquire and process data. Automated sample introduction was achieved using a PAL3 autosampler; this system enables automated GC liner exchange. PAL Sample Control version 3.1 software was used to control the autosampler, and Evolution Workstation 4 software was used to control the GC injector unit (OPTIC) allowing the multifunctional sample introduction techniques.

### Ultrahigh Performance Supercritical Fluid Chromatography–Mass Spectrometry (UHPSFC-MS)

Analysis was undertaken using an acquity ultraperformance convergence chromatograph (UPC^2^, Waters, Wilmslow, U.K.) coupled to a Xevo single quadrupole mass spectrometer (Waters, Wilmslow, U.K.). Supercritical CO_2_ (scCO_2_) was used as the mobile phase with an organic cosolvent. The column used was a Waters acquity BEH 2-EP column (1.7 μm, 3.0 mm × 100 mm) and was kept at a temperature of 40 °C, and the system back pressure was set to 150 bar. The flow rate was 1.5 mL/min and a 2 μL injection volume. The organic modifier used was methanol with 25 mM ammonium acetate, with a 0–40% gradient over 10 min. The makeup flow solvent was methanol with 1% formic acid at a flow rate of 0.450 mL/min.

Positive and negative ion electrospray ionization (ESI) mass spectra were recorded with the following conditions: capillary voltage, 2.5 kV; cone voltage, 20.0 V; extractor, 3.0 V; source temperature, 150 °C; desolvation temperature, 500 °C; and desolvation gas flow, 650 L/h. MassLynx version 4.1 was used to acquire data and subsequently for data processing. Mass spectra were recorded between *m*/*z* 90 and 1000 with a scan duration of 0.2 *s.*

## Results and Discussion

Here, a TD-style sample introduction method (TD 1.0) was developed to afford direct analysis of a fuel filter sample by rapidly heating a small section (∼1 mm^2^) of the fuel filter in the OPTIC GC inlet prior to analysis by GC-MS. Thermal desorption is a process where heating results in volatile and semivolatile compounds desorbing from the matrix.^25,26^ The TD 1.0 procedure works on the assumption that the fuel filter acts as the “adsorbent,” and species deposited onto the fuel filter, during use in a diesel engine, will desorb from the filter upon heating. Once desorbed, the analytes are transferred directly onto the GC column.

This approach is fast and robust and removes the need for extensive sample preparation where a small section of the fuel filter is placed in a sample cup within a GC liner, and this liner is inserted into the OPTIC and the TD process enabled. This direct analysis of the fuel filter prevents the loss of sample information through potential solubility bias in a solvent extraction procedure. This is especially important for complex samples such as diesel, which can contain over 1000 components with differing physicochemical properties and polarities. The TD 1.0 approach is greener than literature methods where multiple sample preparation steps require significant solvent usage.^17,23^ TD 1.0 also addresses potential issues of limited sample quantity as only a very small section of filter is required for analysis.

**Figure 3:**
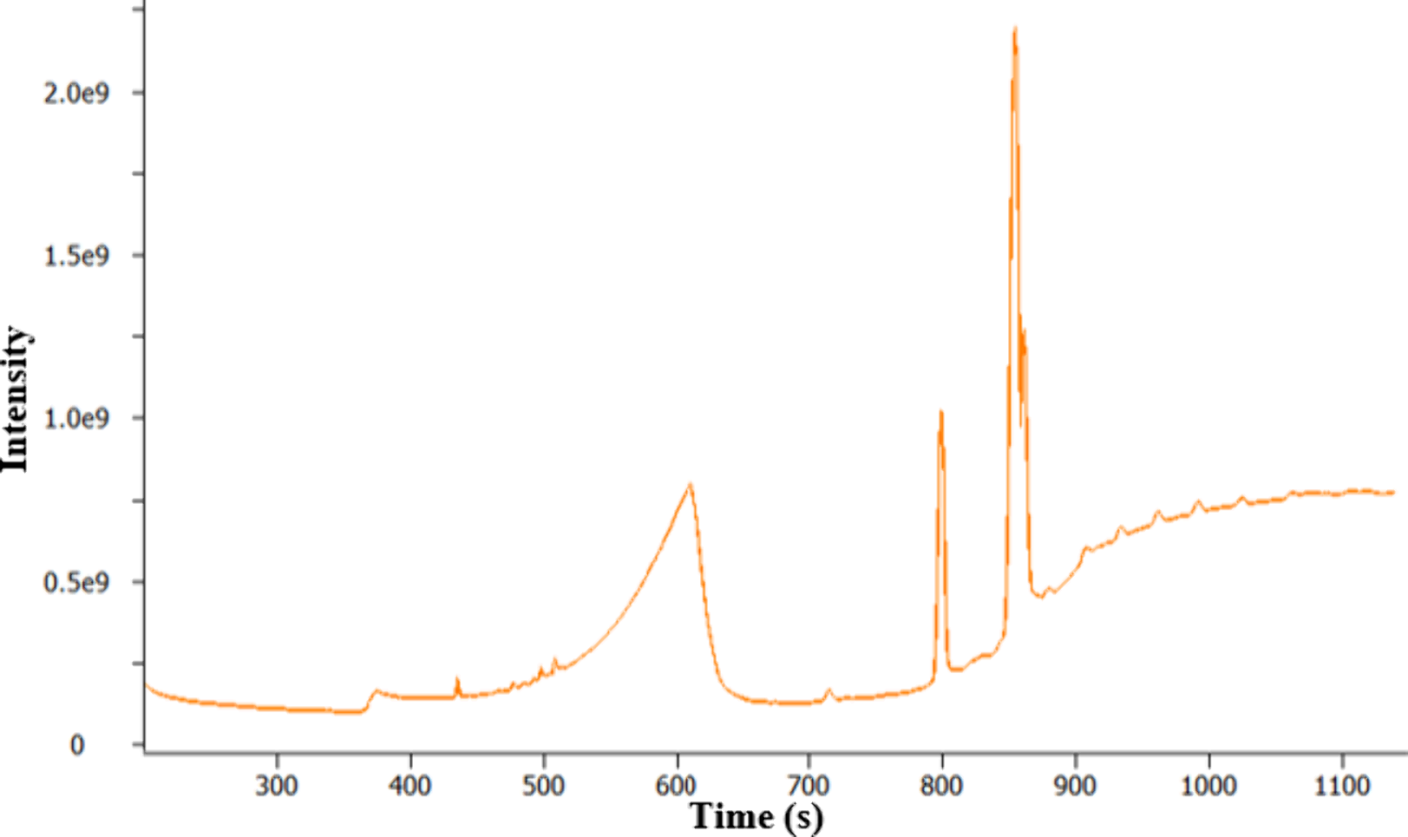
TICC from GC-MS analysis of filter 1 using the TD 1.0 approach.

TD 1.0 was used to analyze a number of fuel filters (1–25), and the results of each filter analysis are listed in Table 1. To demonstrate the efficacy and adjustments of the new approach, the data from two filters (filters 1 and 2) are shown. Some fuel filters were visibly soiled whereas others appeared clean. GC-MS analysis of “filter 1” (visibly clean filter) is shown in Figure 3. The peaks in the total ion current chromatogram (TICC) at retention time (*t*_R_) 799, 854, and 861 s were identified as FAME species by comparison of their mass spectra with the NIST EI mass spectral library. A similarity index (SI) value is algorithmically determined to numerically assess how similar the measured mass spectrum is with a library match; this is a number between 1 and 999—the higher the number, the closer the mass spectrum matches the library entry.^27^ NIST Standard reference database users guide states that an SI between 900 and 999 indicates an excellent match, 800 to 900 is a good match, 700 to 800 is a fair match, and anything less than 600 is a poor match.^28^

**Table 1:**
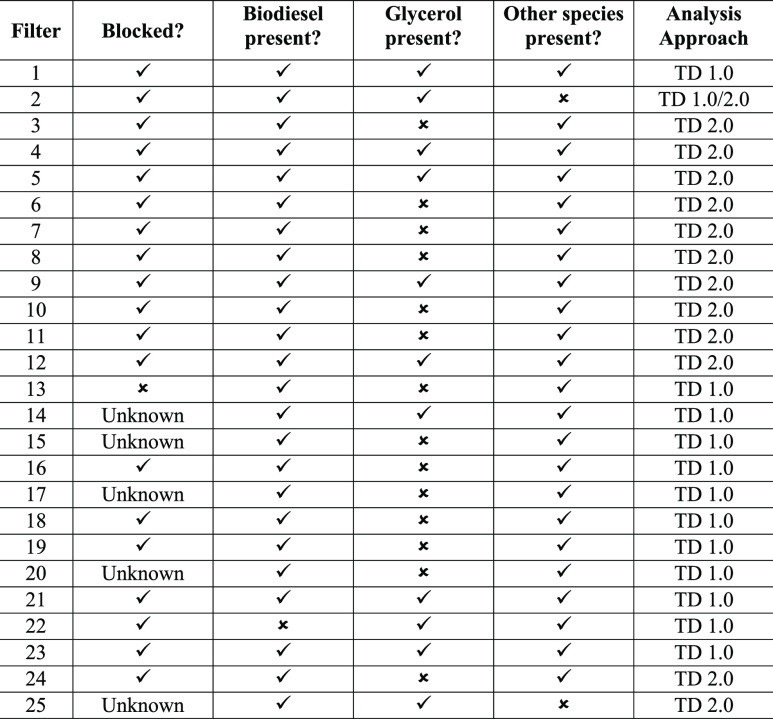
Blocked Fuel Filter Analysis Results from 25 Different Filters and the Method Used To Analyze the Samples^a^

a Other species included known problematic components, such as FFAs, MAGs, or FAME oxidation products.

**Figure 4:**
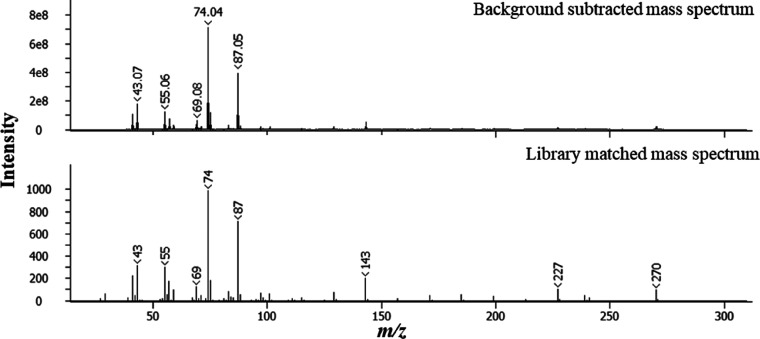
Background subtracted 70 eV electron ionization mass spectrum and associated library match mass spectrum for peak with *t*_R_ 799 s corresponding to C16:0 FAME.

**Figure 5:**
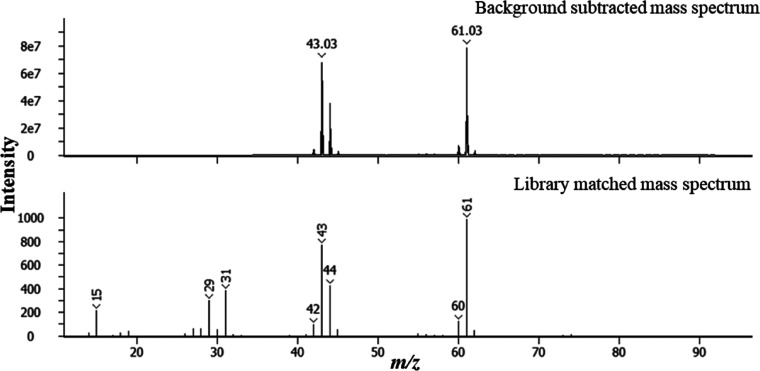
Background subtracted 70 eV electron ionization mass spectrum and associated library match mass spectrum for peak between t_R_ 366 and 650 s corresponding to glycerol.

Figure 4 shows the mass spectrum for the peak at *t*_R_ 799 s, which gives an SI of 874 for C16:0 FAME (hexadecanoic acid methyl ester, C_17_H_34_O_2_). Other peaks corresponding to biodiesel (FAMEs) were identified following the same protocol. A large, asymmetrical peak is present between *t*_R_ 366 and 650 s. The mass spectrum for this peak (*t*_R_ = 611 s) is shown in Figure 5 and corresponds to the library match of glycerol (SI 957). This finding is consistent with other studies of plugged fuel filters where the presence of glycerol was identified.^18,19^ GC-MS is an advantageous technique as it is a common instrument found in analytical laboratories. More complex instrumentation such as comprehensive two-dimensional gas chromatography-mass spectroscopy (GCxGC-MS) presents a powerful tool for the analysis of complex mixtures. However, these instruments are expensive and can incur high running costs; therefore, GCxGC-MS is not widely accessible across analytical laboratories. Using TD 1.0 in combination with GC-MS makes the approach widely applicable as the method is more readily transferable to other laboratories.

Glycerol is a byproduct of the manufacturing process of biodiesel, where triacylglycerides (TAGs) obtained from plant or animal material are transesterified to produce FAMEs. Glycerol can be used as an indicator of biodiesel quality as it has been linked with deposit formation and issues with fuel filterability.^20^ Therefore, glycerol should be removed from the final product, and this is often achieved by centrifugation.^29^ Free glycerol levels in biodiesel are limited to 0.02% mass in both EN 14214 and ASTM D6751,^24^ and this is monitored using test methods EN 14105 in Europe and D6584 in the USA.^15^ Despite these tests, fuels containing glycerol within these limits have been linked with incidences of fuel filter plugging.^17^

Heiden et al. identified significant imprecision in test methods for free glycerol content that may allow approval of substandard fuel. In addition to this imprecision, it was determined that glycerol forms insoluble agglomerates below the limits set for glycerol in fuel.^20^ They also suggested that the presence of water in the fuel can further worsen the solubility of glycerol in the fuel due to the formation of glycerol/H_2_O “heterophases;” in addition, it was noted lower temperatures would further lower the solubility of glycerol.

Identification of this analyte in real-world plugged fuel samples suggests its presence may lead to blockages; this may have resulted from a multitude of issues. Poor handling of the fuel may have allowed water to enter the system and reduce the solubility of glycerol; alternatively, biodiesel is a hygroscopic material, so it may absorb water from the atmosphere.^19^ Cumulative effects may have led to the slow buildup of glycerol in storage tanks overtime allowing the formation of large insoluble agglomerates.^20^ Additionally, operation at low temperatures may have worsened the solubility of glycerol. These potential reasons for blockage formation could have occurred as part of a synergistic process or independently.

**Figure 6:**
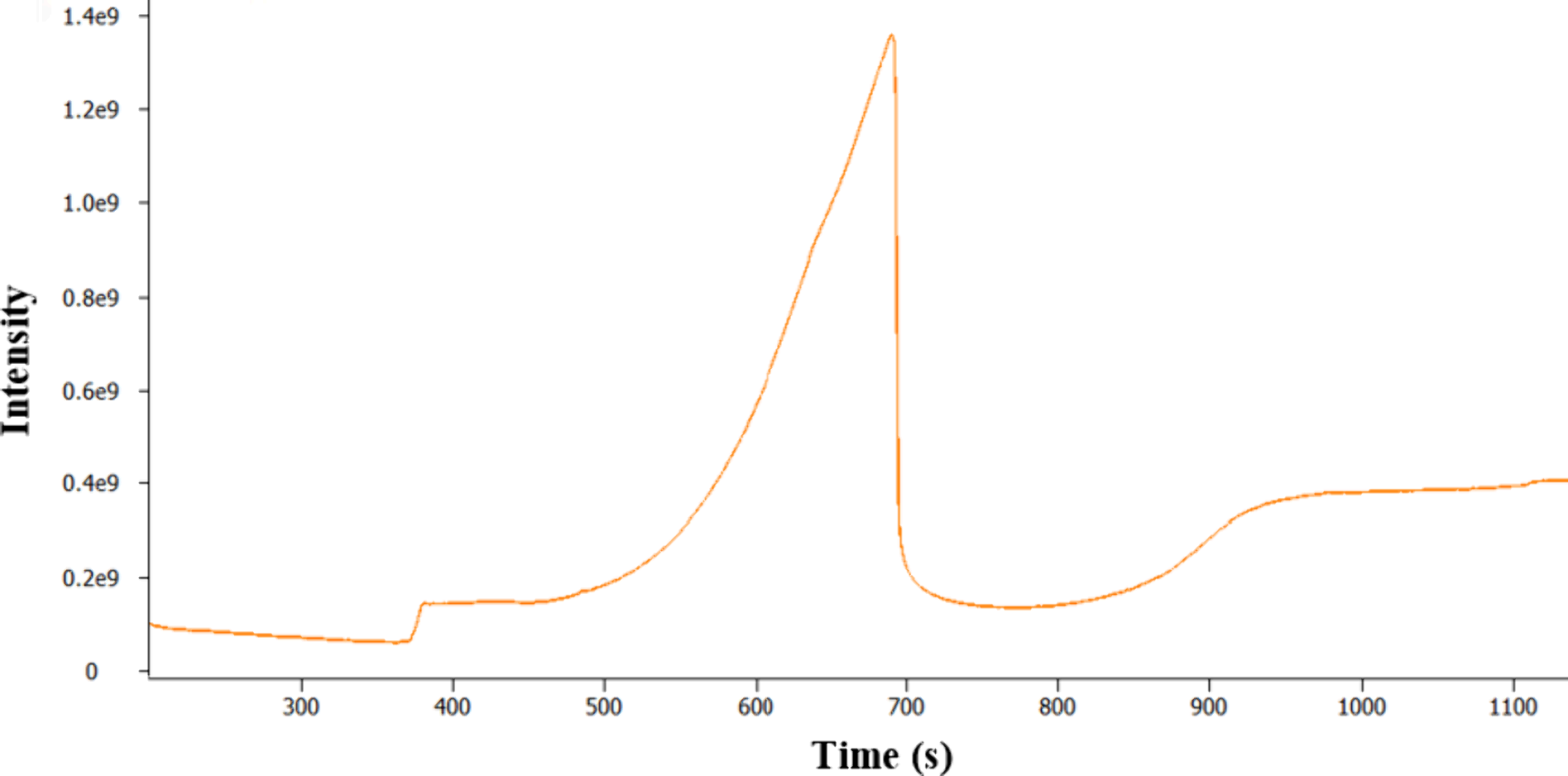
TICC from TD 1.0 GC-MS analysis of a glycerol standard sample and the characteristic asymmetrical peak present between 370 and 700 s.

To confirm the presence of glycerol in the filter sample, a putative glycerol sample was analyzed and the results are shown in Figure 6, where a large asymmetrical peak is observed between *t*_R_ 370 and 700 s. This is a similar peak shape to the peak assigned as glycerol in the analysis of filter 1 shown in Figure 3. This similarity in peak shape provides further confidence in the identification of glycerol. The slight differences between the retention times can be attributed to the potential difference in the concentration between the two samples. Confidence of assignment was further enhanced through high-resolution, accurate mass measurement of the EI MS (data not shown).

**Figure 7:**
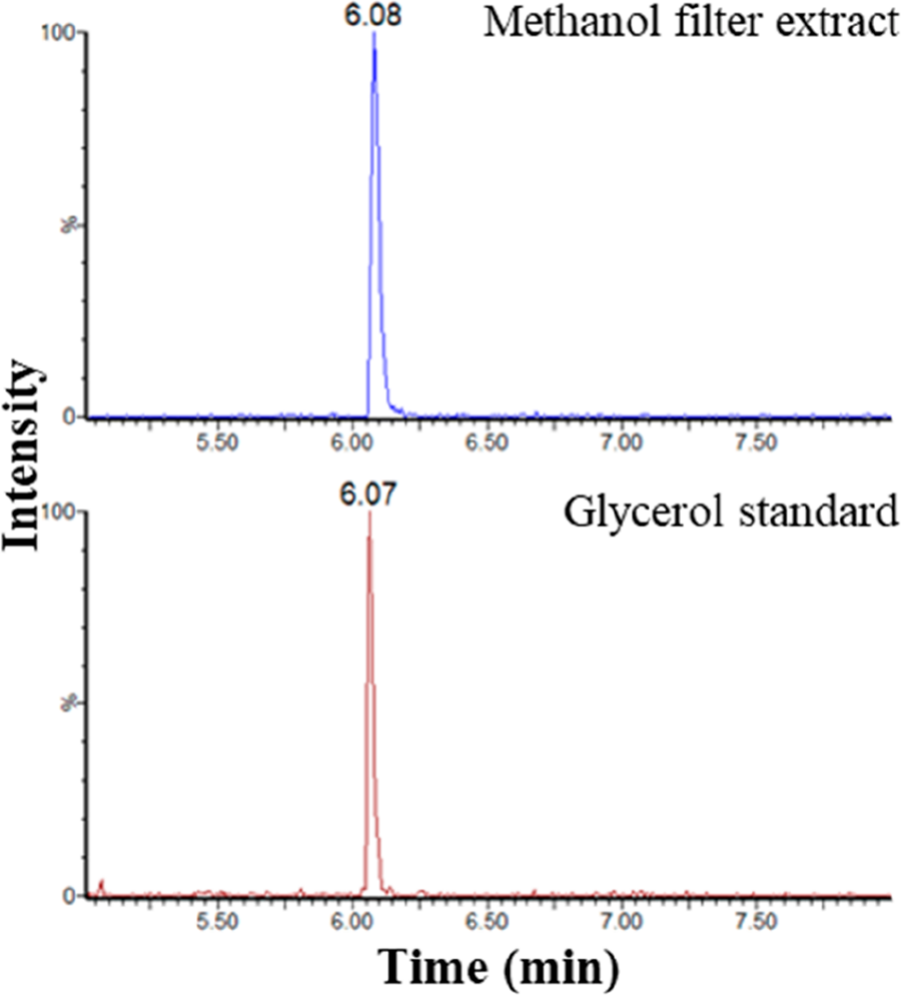
UHPSFC-MS negative ion ESI RICCs for [M – H]^−^ for *m*/*z* 91 for the glycerol standard and the methanol fuel filter extract.

**Figure 8:**
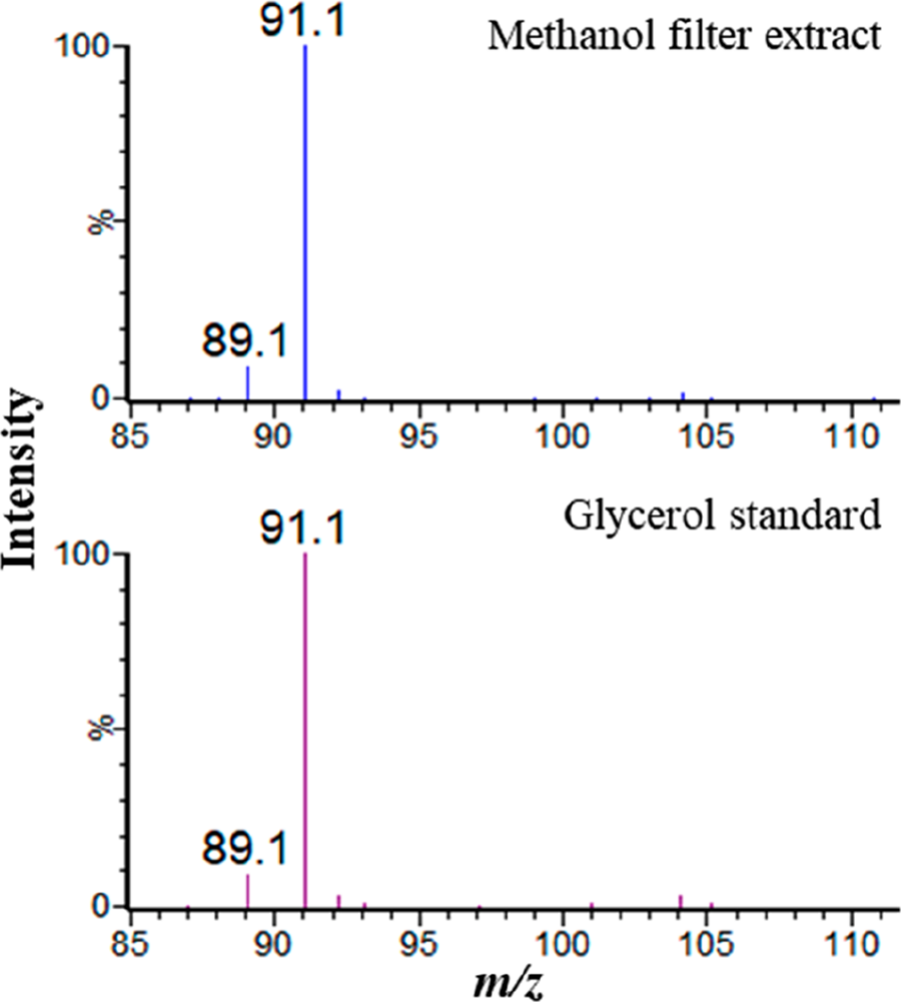
Negative ion ESI spectrum for glycerol standard (bottom) and methanol filter extract (top) UHPSFC-MS analysis.

**Figure 9:**
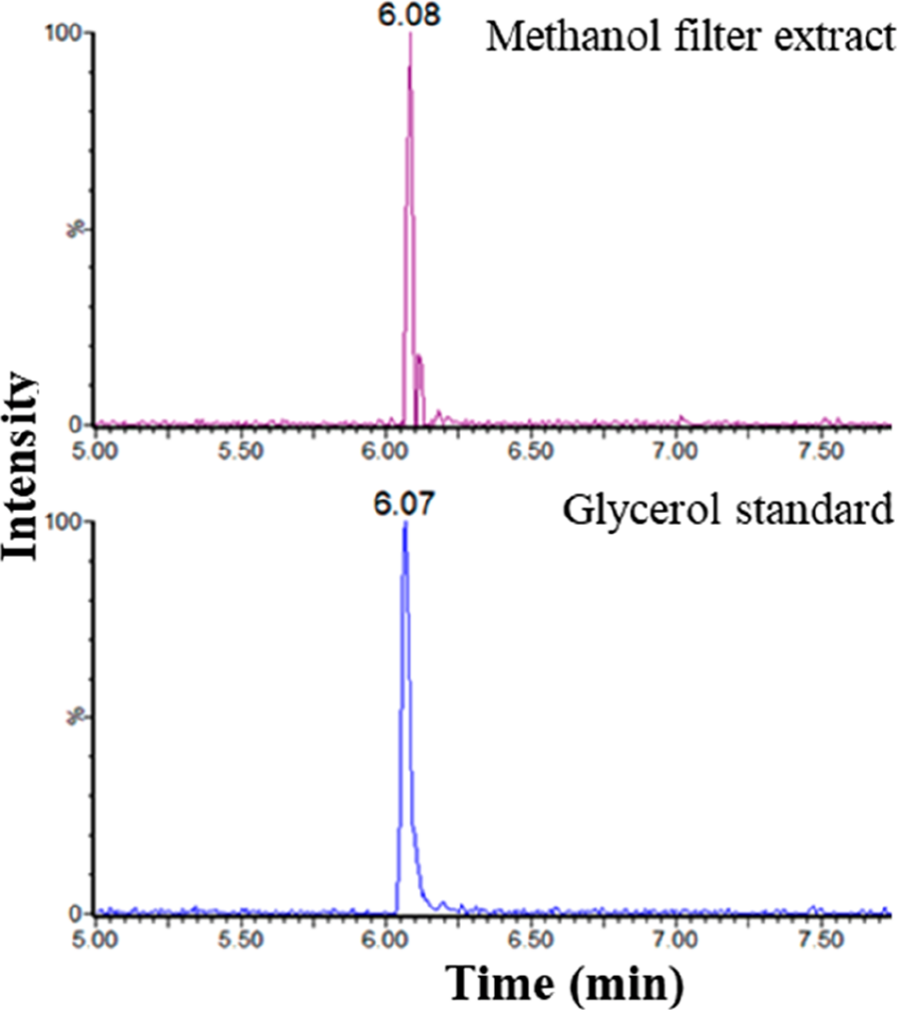
UHPSFC-MS positive-ion ESI RICCs for [M + Na]^+^ and *m*/*z* 115 for the glycerol standard and the methanol fuel filter extract.

Methanol extracts from the filter papers were also analyzed by UHPSFC-MS, and both positive and negative ion ESI spectra were recorded. A putative glycerol sample was also analyzed. Reconstructed ion current chromatograms (RICCs) for the [M – H]^−^ glycerol ion (*m*/*z* 91) suggested glycerol may be present in the methanol extract of the filter (see Figure 7). The mass spectrum can be seen in Figure 8. This is corroborated by RICCs for the [M + Na]^+^ glycerol ion (*m*/*z* 115) as shown in Figure 9. The possible identification of glycerol in the methanol extract of this filter using UHPSFC-MS analysis supports the data from the GC-MS analysis of this extract, where the presence of glycerol is also indicated.

The analysis shows glycerol has a *t*_R_ of 6.07 min, which is similar to the suspected glycerol peak in the methanol extract of filter 2 which had a *t*_R_ of 6.08 min. The peak shape between both samples is also similar, a sharp peak shape with slight peak tailing. These similarities in retention time and peak shape between the glycerol standard and the fuel filter give further confidence that glycerol is present in the filter.

**Figure 10:**
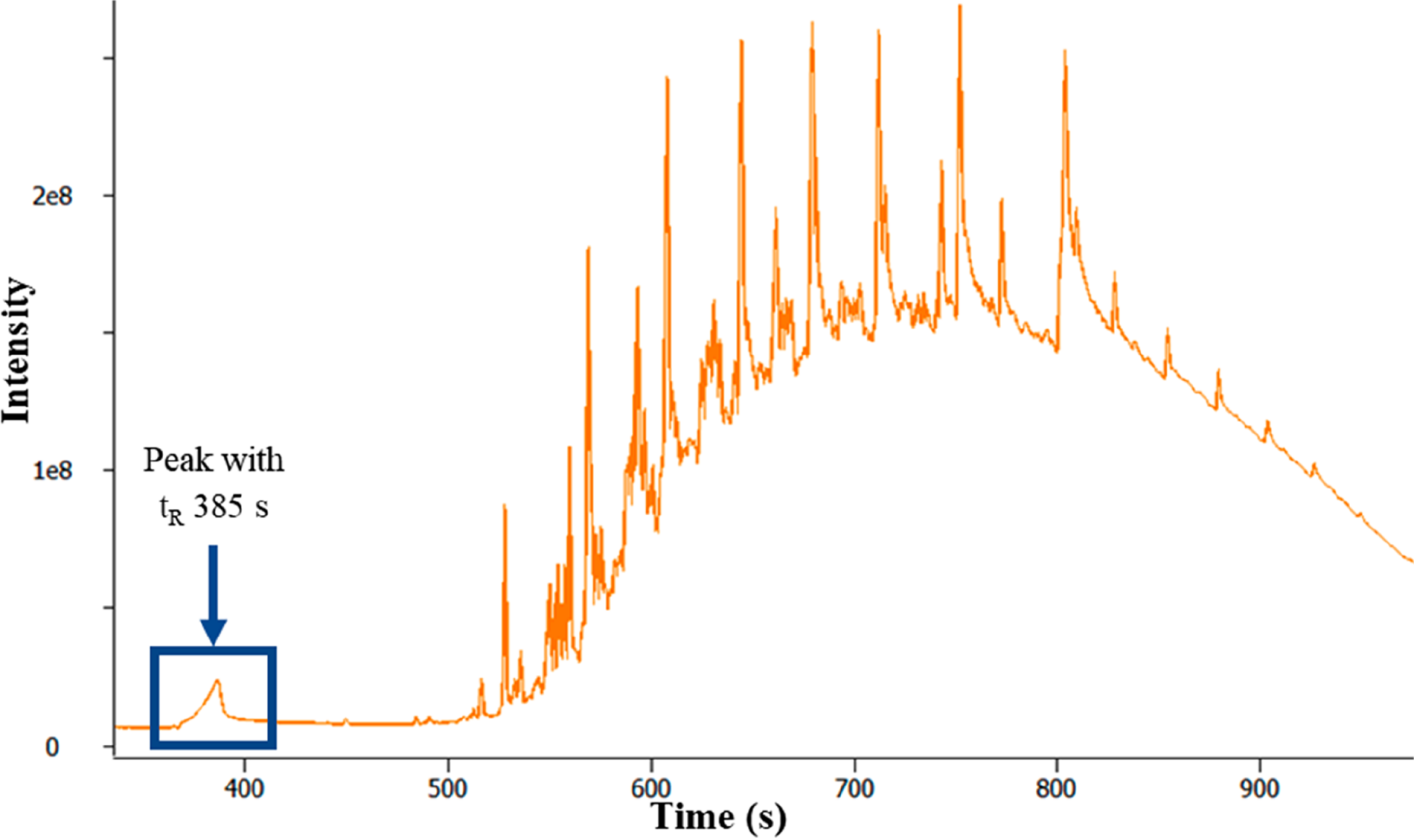
TICC GC-MS analysis of a heavily soiled fuel filter using TD 1.0.

Analysis of more heavily soiled fuel filters using TD 1.0 often resulted in an overloaded chromatogram (see Figure 10) due to the excess fuel matrix. Overload can result in changes to peak shapes (loss of peak symmetry, peak broadening, etc.), changes to retention time, loss of resolution, and corruption of isotope patterns in the mass spectrum. This can lead to a loss of information about the sample and reduced confidence in the identification of analytes. In these cases, a fast and simplified sample clean-up procedure was developed and used to reduce the levels of fuel matrix introduced into the GC-MS to prevent overload and simplify the data. This adjustment to the analysis approach was termed TD 2.0.

Despite the overloaded TICC shown in Figure 10, valuable information can still be extracted from the data. In this specific filter analysis, a small peak in relation to the fuel matrix is observed at *t*_R_ 385 s; this was consistent with previous data determining the presence as glycerol (SI 875).

To improve confidence in this identification and simplify the data, the TD 2.0 approach was employed. Different solvents with different solubilizing properties were used to wash the fuel filters, and the solvent wash removed certain analytes from the filter depending on their polarity and solubility with the solvent. Fuel filters were soaked in different solvents for 15 min and then removed; the TD 2.0 procedure yielded solvent-washed filter-paper samples and solvent extracts for analysis. The solvent extracts were analyzed using liquid injection GC-MS as well as by complementary analysis using UHPSFC-MS.

The TD 1.0 approach is time efficient and eliminates solvent use; therefore, it is important that TD 2.0 is similarly time efficient and uses minimal solvent. The method used in this work is straightforward, takes a maximum of 20 min, and uses a small volume of solvent (∼6 mL per sample), and this is a significantly shorter and more sustainable sample preparation method than those used by Heiden et al. and Csontos et al.^17,23^ DCM and methanol were the solvents used, and the method is described in the Experimental Section of this paper. The results of filter 2 are shown to demonstrate the results of the TD 2.0 approach. The resulting samples used for analysis were a DCM extract, a methanol extract, a DCM-washed filter 2 section, and a methanol-washed filter 2 section.

**Figure 11:**
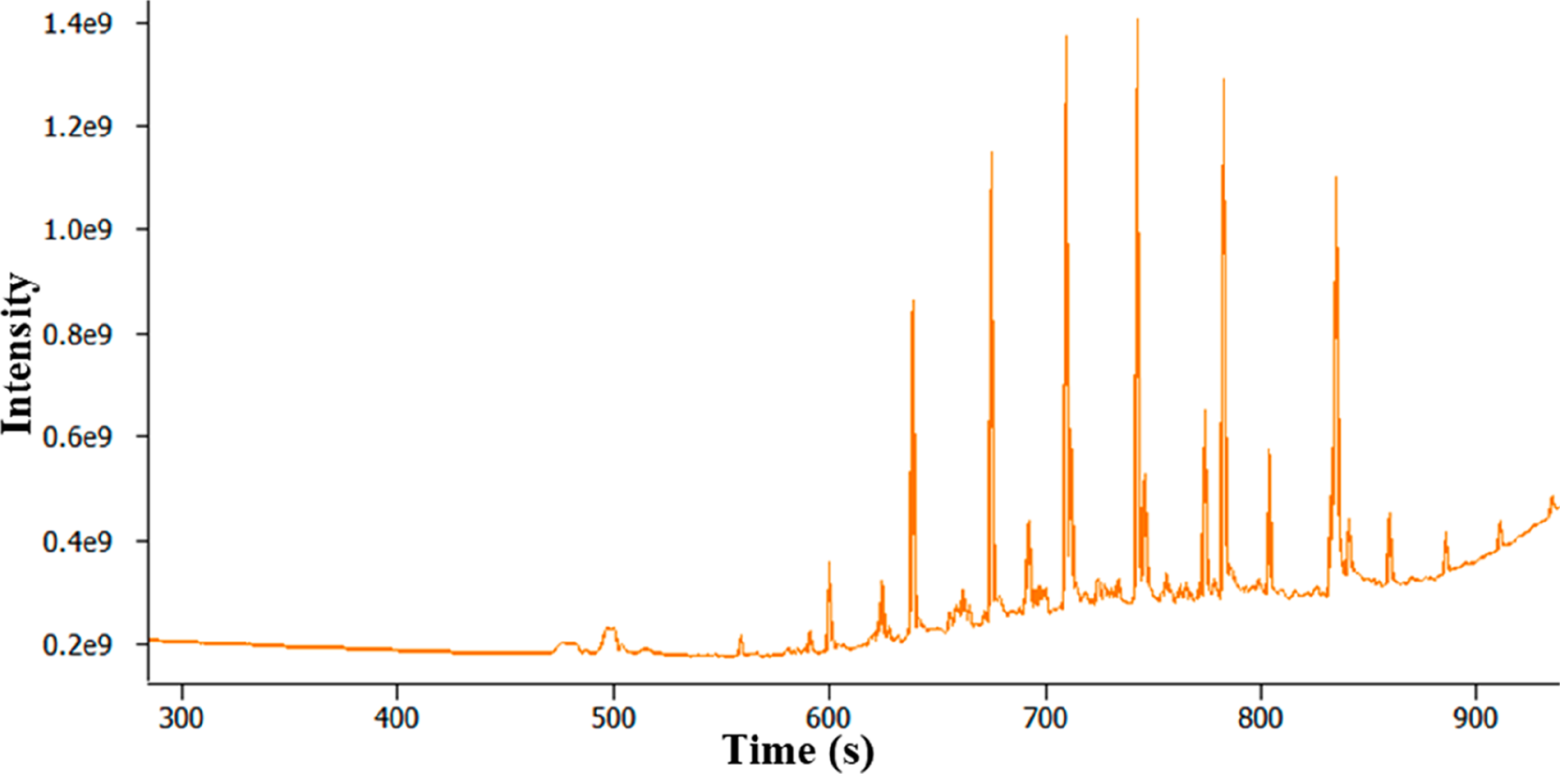
TICC of the DCM extract of filter 2.

GC-MS analysis of the DCM extract of filter 2 is shown in Figure 11 and reveals the presence of diesel matrix in the filter sample, and diesel is highly soluble in DCM. An alkane series is present, and the corresponding peaks are seen at high intensities. This analysis also shows the presence of biodiesel; peaks with t_R_ 783 and 835 s are identified as C16:0 and C18:1 FAME with SIs of 962 and 958, respectively.

**Figure 12:**
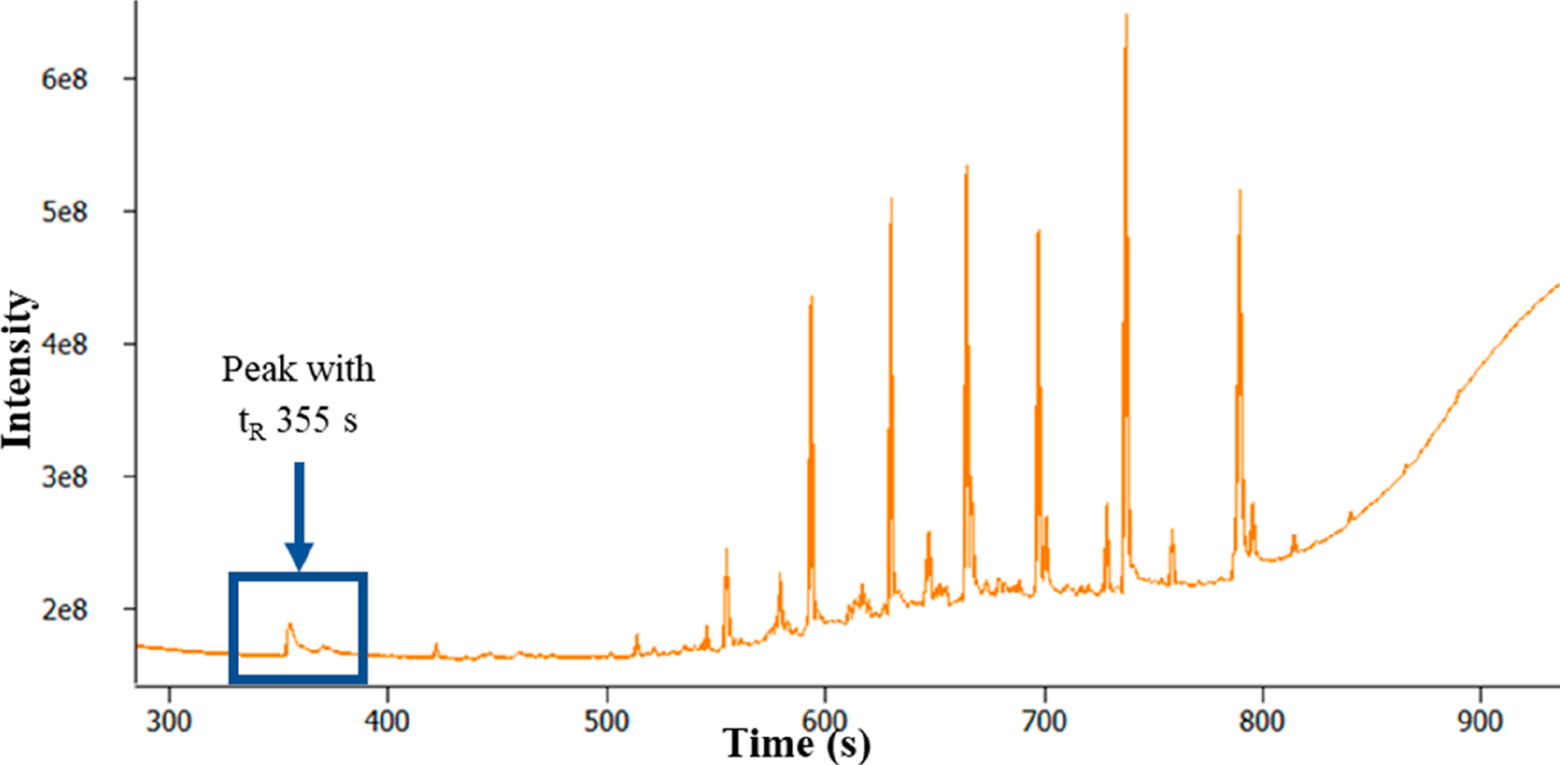
TICC of the methanol extract of filter 2.

**Figure 13:**
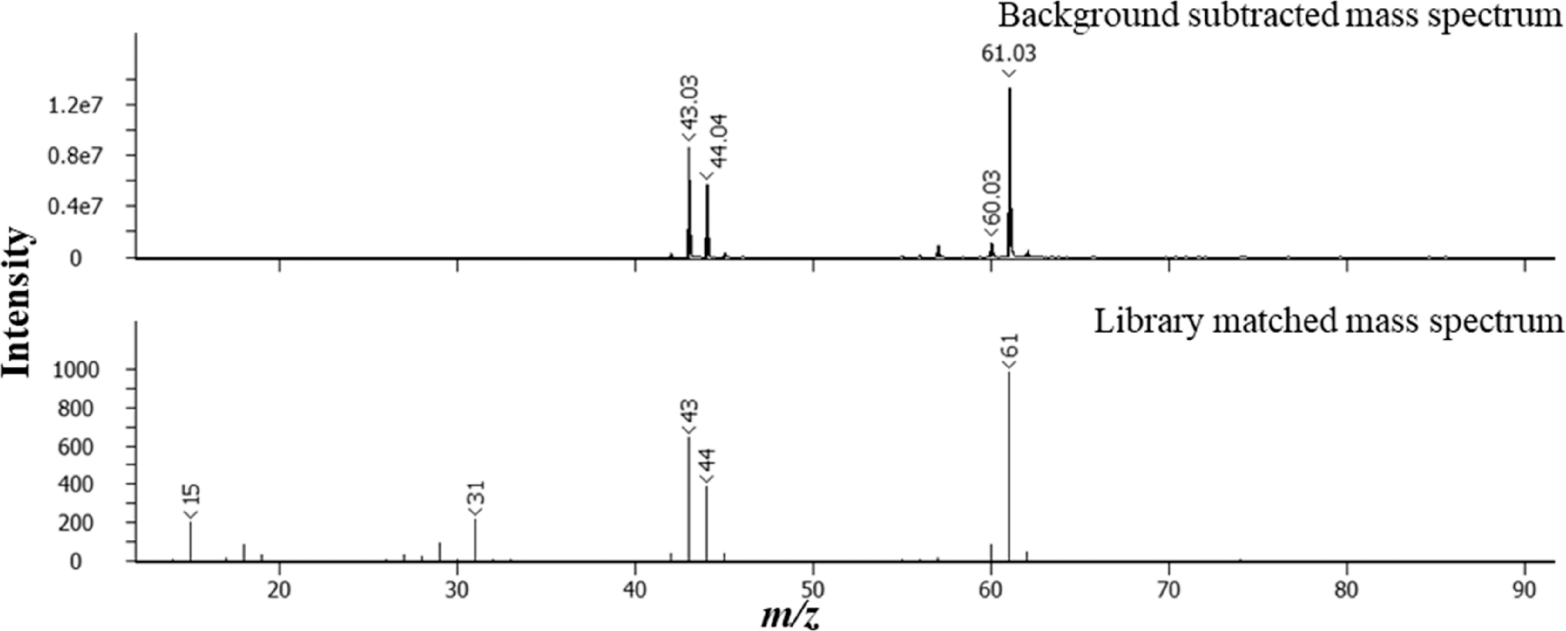
70 eV electron ionization mass spectrum for peak with *t*_R_ 355 s and a library match.

The methanol extract of filter 2 shows similar information to the DCM extract with a smaller portion of the diesel matrix present (see Figure 12). However, in addition to the expected diesel analytes, the methanol extract also shows a peak with *t*_R_ = 355 s in the TICC that is not seen in the DCM extract data. The EI MS associated with this peak, shown in Figure 13, gives a library match SI of 892 for glycerol.

**Figure 14:**
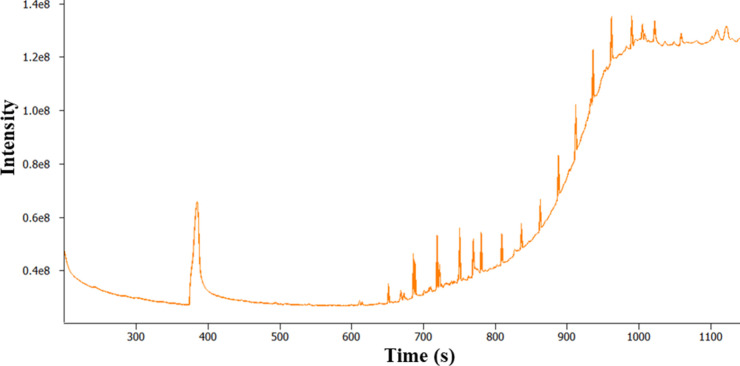
TICC of the DCM washed filter 2 using TD-style sample introduction.

**Figure 15:**
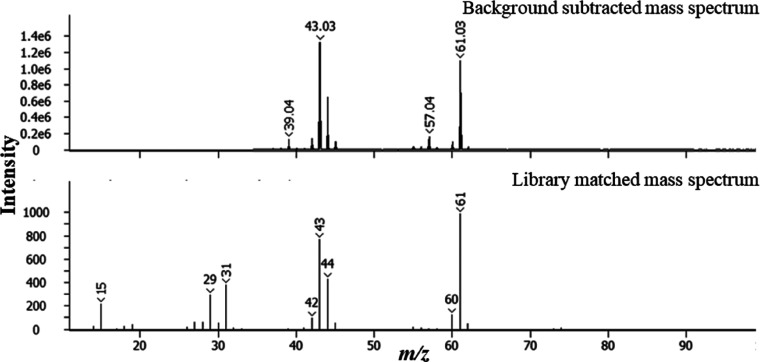
Background subtracted 70 eV electron ionization mass spectrum and associated library match spectrum for peak with *t*_R_ 403 s.

The GC-MS data obtained after the sample washing approach were used to produce simplified chromatograms and to improve the detection of the polar analyte (glycerol). TD analysis of the DCM-washed filter-paper analysis shows that most of the diesel matrix was washed away during the washing process (see Figure 14). This was expected as GC-MS analysis of the DCM solvent extract indicated that a large quantity of diesel matrix was present in the sample. There is a large nonsymmetrical peak with *t*_R_ 403 s, and the associated EI MS gives a library match for glycerol with an SI of 841 (see Figure 15).

This corroborates data obtained from the GC-MS analysis of the corresponding methanol solvent extract, which shows the presence of glycerol.

There was no indication of whether glycerol was present in the methanol-washed filter paper when analyzed using a TD-style sample introduction. This was expected since glycerol is soluble in methanol and would have been removed during the washing stage. TD 2.0 for the analysis of fuel filters yields comparable results to the previous literature and fast identification of glycerol; in this case, the approach to analysis enables results to be obtained in 40 min from sample arrival to GC-MS results.

Although the chromatographic peak shape for glycerol (as seen in Figure 14) is poor, this peak shape can be indicative of the presence of a polar species. In addition, orthogonal analysis of the putative compound and the solvent extracts obtained from TD 2.0 analysis of filter 2 were undertaken using UHPSFC-MS where glycerol was also identified.

The presence of glycerol in this real-world sample suggests a biodiesel-related issue. TD 1.0 and TD 2.0 might help identify other biodiesel-related problematic material such as unreacted material from FAME manufacture including triacylglycerides, diacylglycerides, and monoacylglycerides (TAGs, DAGs, and MAGs), as well as free fatty acids (FFAs) or FAME oxidation products for example. To test the efficacy of the TD style sample introduction GC-MS analysis method for the detection of other known problematic species, a standard sample of free fatty acid (FFA) was prepared. FFAs may be present in the fuel as a lubricity improver additive, or conversely, FFAs could be a byproduct of biodiesel manufacture.^15^ FFAs have been identified as a potential problematic material, and they have been linked with metal carboxylate formation resulting in plugging incidences in fuel filters and internal diesel injector deposits.^30^

**Figure 16:**
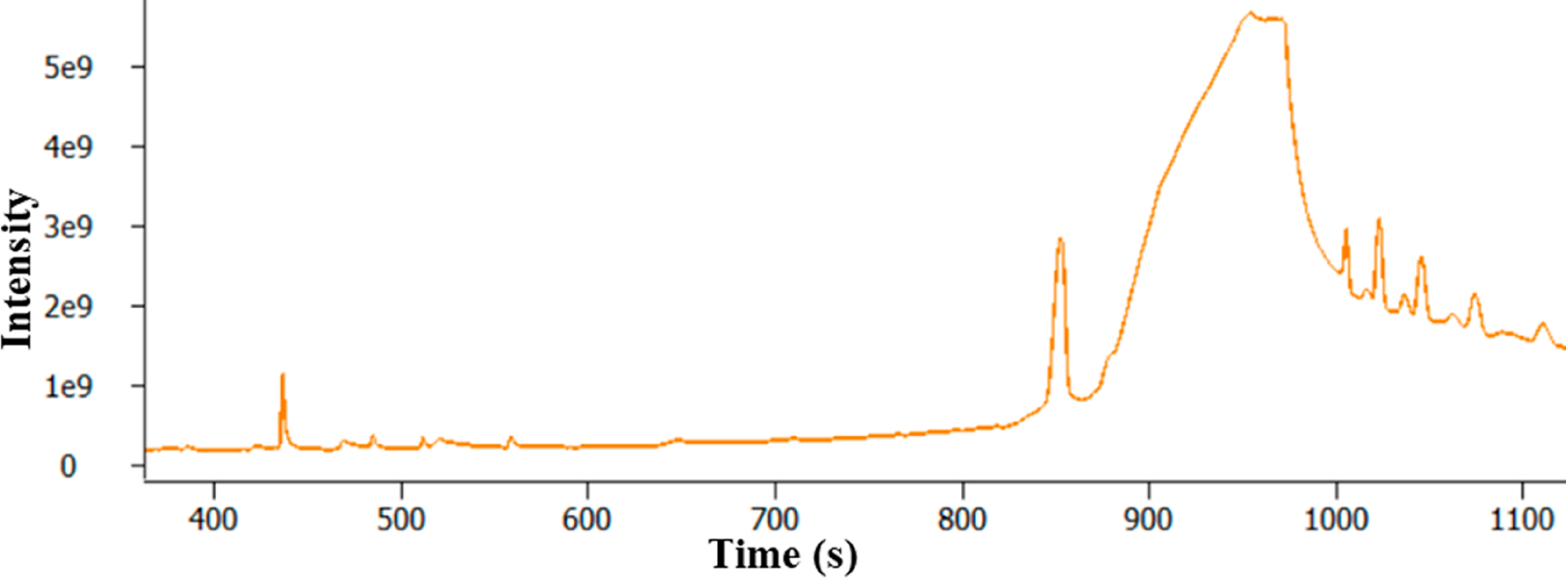
TICC of oleic acid using a TD-style sample introduction.

A 500 μg/mL sample of oleic acid was prepared in DCM, 100 μL of this was pipetted into a microvial, and the solvent was left to evaporate, leaving only 50 μg of oleic acid in the vial. This process was repeated with a DCM blank for comparison. This sample was then analyzed using a TD-style sample introduction with a 10:1 split. The resulting chromatogram shows a large peak with *t*_R_ 960 s (see Figure 16). The corresponding mass spectrum library hit gives an SI of 960 for oleic acid (cis-9-octadecanoic acid, C_18_H_34_O_2_. C18:1 acid). The peak with *t*_R_ 852 s corresponds to a C18:1 FAME, and this may be present in the standard oleic acid sample as an impurity.

In addition to the identification of the C18:1 FFA, other biodiesel-related material has also been indicated as present in the sample, such as smaller chain FFAs and oxidation products including aldehydes. A TAG sample was also analyzed, and thermal degradation products indicating the presence of a TAG were identified, such as FFAs (data not shown). Notably, glycerol was not present in the TD style sample introduction GC-MS analysis of a TAG sample; this may suggest that the presence of glycerol is not formed by thermal degradation of TAGs, DAGs, or MAGs. This signifies that this analysis method is suitable for detecting biodiesel-related species present in a fuel filter sample.

The TD-style analysis approach was used on multiple different filter-paper samples collected from different filter-plugging scenarios across the world. Some of the results from these analyses can be seen in Table 1, glycerol was identified in 11 of these filters, and, of these 11 filters, biodiesel was present in 10 of them. In addition to glycerol, other problematic material was also found in 23 of the 25 samples; these included FFAs and monoacylglycerides (MAGs). Thirteen of the 25 samples were analyzed using TD 1.0 which meant no prior sample preparation was necessary; this allowed rapid analysis. In the 12 cases where the TD 2.0 approach was necessary, analysis was still fast due to the quick and simple sample preparation method employed.

## Conclusions

The new approach to the analysis of diesel-engine fuel filters discussed in this paper allows rapid and unbiased analysis of filters, enabling key information about the samples to be uncovered swiftly and simply. TD 1.0 allows the unbiased analysis of a fuel filter as it can remove the potential solubility bias involved in sample preparation. The analysis of heavily soiled fuel filters called for a straightforward and fast sample clean-up procedure; this was developed with a focus on sustainability and removing potential solubility bias. This was achieved using multiple solvents during the sample cleanup procedure and ensuring that only a minimal volume of solvent was used. The sample cleanup process used is faster than the methods described in the previous literature and produces comparable results. The TD 2.0 approach enables the analysis of the raw and washed fuel filters as well as solvent extracts; therefore, a holistic picture of the fuel filter can be built by simplifying complex data, thus easing data processing and interpretation.

Overall, the new method utilizes complementary sample preparation and introduction techniques compatible with different chemical properties, ensuring comprehensive analysis of a fuel filter sample. This is essential in the identification of unknowns in complex samples, such as diesel fuel filters, as the chemical nature of the problematic components is not always apparent. This new approach can be used in conjunction with alternative complementary techniques such as UHPSFC-MS to improve confidence in the assignment of problematic material identified within the fuel filter sample.

These techniques have proven useful in the detection of problematic components; the data obtained suggest that glycerol is present in multiple different filter samples. Glycerol is a known problematic component and, therefore, may contribute to fuel filter plugging. As well as glycerol, other known problematic components have also been identified within the fuel filters. Using the approaches discussed in this paper allows the rapid analysis of fuel filters and, therefore, fast identification of any problematic analytes. Identification of these analytes is useful in developing mitigation strategies to help prevent fuel filter blockages in the future.

## ABBREVIATIONS

DAG: diacylglyceride
DCM: dichloromethane
EI: electron ionization
ESI: electrospray ionization
FAME: fatty acid methyl ester
FFA: free fatty acid
FID: flame ionization detector
FTIR: Fourier transform infrared
GC-MS: gas chromatography–mass spectrometry
GCxGC-MS: comprehensive two-dimensional gas chromatography–mass spectrometry
MAG: monoacylglyceride
NIST: the National Institute of Standards and Technology
RICC: reconstructed ion current chromatogram
scCO_2_: supercritical carbon dioxide
SI: similarity index
TAG: triacylglyceride
TD: thermal desorption
TICC: total ion current chromatogram
UCO: used cooking oil
UHPSFC-MS: ultrahigh performance supercritical fluid chromatography–mass spectrometry
USA: United States of America
XRF: X-ray fluorescence
